# Ecological impact assessment of climate change and habitat loss on wetland vertebrate assemblages of the Great Barrier Reef catchment and the influence of survey bias

**DOI:** 10.1002/ece3.7412

**Published:** 2021-03-24

**Authors:** Adam D. Canning, Nathan J. Waltham

**Affiliations:** ^1^ Centre for Tropical Water and Aquatic Ecosystem Research (TropWATER) James Cook University Townsville Qld Australia

**Keywords:** climate change, Great Barrier Reef, species distribution modeling, survey bias, Wetland loss

## Abstract

Wetlands are among the most vulnerable ecosystems, stressed by habitat loss and degradation from expanding and intensifying agricultural and urban areas. Climate change will exacerbate the impacts of habitat loss by altering temperature and rainfall patterns. Wetlands within Australia's Great Barrier Reef (GBR) catchment are not different, stressed by extensive cropping, urban expansion, and alteration for grazing. Understanding how stressors affect wildlife is essential for the effective management of biodiversity values and minimizing unintended consequences when trading off the multiple values wetlands support. Impact assessment is difficult, often relying on an aggregation of ad hoc observations that are spatially biased toward easily accessible areas, rather than systematic and randomized surveys. Using a large aggregate database of ad hoc observations, this study aimed to examine the influence of urban proximity on machine‐learning models predicting taxonomic richness and assemblage turnover, relative to other habitat, landscape, and climate variables, for vertebrates dwelling in the wetlands of the GBR catchment. The distance from the nearest city was, by substantial margins, the most influential factor in predicting the richness and assemblage turnover of all vertebrate groups, except fish. Richness and assemblage turnover was predicted to be greatest nearest the main urban centers. The extent of various wetland habitats was highly influential in predicting the richness of all groups, while climate (predominately the rainfall in the wettest quarter) was highly influential in predicting assemblage turnover for all groups. Bias of survey records toward urban centers strongly influenced our ability to model wetland‐affiliated vertebrates and may obscure our understanding of how vertebrates respond to habitat loss and climate change. This reinforces the need for randomized and systematic surveys to supplement existing ad hoc surveys. We urge modelers in other jurisdictions to better portray the potential influence of survey biases when modeling species distributions.

## Graphical abstract

We show that, when using a large citizen science biodiversity, modeling the distributions of vertebrate species richness and assemblages within Australia's Great Barrier Reef catchment is heavily influenced by the biased collection of data nearest to cities. While citizen science can be a useful supplement to existing data, it should not be a replacement for randomized and systematic ecological surveys. Failing to understand how species change with wetland habitat extent and climate means that it will be difficult for managers to understand the consequences of climate change or outcomes expected from wetland restoration.

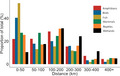

## 1 INTRODUCTION

Habitat loss and climate change are the two largest human impacts on ecosystems throughout the Anthropocene (Bellard et al., 2012; Johnson et al., 2017; Pecl et al., 2017). Globally, continued habitat loss alone is projected to drive approximately 1,700 vertebrate species to extinction by 2070 (Powers & Jetz, 2019). Wetlands are among the most vulnerable of these ecosystems, with a global assessment predicting that 100% of wetlands are likely or highly likely to suffer the most from habitat loss and fragmentation exacerbated by climate change, compared with rainforests as second most impacted ecosystems at 45.3% (Segan et al., 2016). Approximately 10% of global animal biodiversity is associated with freshwater ecosystems, which occupy <1% of Earth's surface (Dudgeon, 2019). Given the scarcity of freshwater, the majority of freshwater ecosystems experience high human exploitation, primarily from habitat conversion to agriculture and urban dwellings, habitat degradation, pollution, climate change, water abstraction for irrigation, and dams (Dudgeon, 2019). Wetlands are areas of permanent or periodic/intermittent inundation, with water that is static or flowing fresh, brackish, or salt, including areas of marine water the depth of which at low tide does not exceed 6 meters—it is this definition we will use in this study (Environmental Protection Agency, 2005; Ramsar Convention Secretariat, 2016). Wetlands hold high biodiversity value and provide critical functions, such as reducing nutrient and sediment runoff, dampen the impact of floods, and provide carbon abatement (Li et al., 2018; Tomer et al., 2009).

Wetlands deliver many values or services, but not all provide the same values or services: One wetland may be primarily valued for its natural amenity, while another is considered more important for biological productivity or cultural values (Maltby & Acreman, 2011; Tomer et al., 2009). Effective environmental management involves balancing multiple values that achieve the best outcomes economically, socially, and environmentally (Martin et al., 2018; Moomaw et al., 2018). Understanding how the diversity and distribution of wetland‐affiliated species may be altered by stressors, such as habitat loss and climate change, is essential for the effective management of biodiversity values and minimizing unintended consequences (e.g., Finlayson et al., 2006; Halse et al., 2004; Junk et al., 2006).

With the rapid accumulation of environmental and species distribution data, and an increasing ability to recruit a plethora of new modeling methods, interest in species distribution modeling has been rising at a rapid pace. Species distribution models (SDMs) are proving useful in species and ecosystem management (Death, 2015; Pecchi et al., 2019; Villero et al., 2017). However, regardless of the wealth of data or modeling techniques, the old adage of “garbage in, garbage out” is still a fundamental challenge for modelers and managers (Rose & Fischer, 2011; Zuckerberg et al., 2011). Poor data cloud the ability to effectively encapsulate relationships and accurately predict beyond the range of input data (i.e., extrapolate to new scenarios). In the absence of rigorous ecological assessments, consortium databases that combine surveys from many small studies and citizen science observations are often used (Fletcher et al., 2019; Zuckerberg et al., 2011). While the data may still be the best available, citizen science observations and ad hoc surveys can be often biased toward easy to access areas (e.g., roads or walking tracks), lack systematically derived absence records, and may fail to adequately represent remote or inaccessible ecosystems (Boakes et al., 2010; Fletcher et al., 2019; Piccolo et al., 2020). The extent to which this bias blurs knowledge on species distribution patterns largely depends on the spread of urban centers and the proximity of ecosystems to those urban centers. Methods are available to minimize sampling bias though they cannot account for a lack of data outside the commonly surveyed environmental range and, therefore, are not a panacea for poor survey data (e.g., Fletcher et al., 2019; Robinson et al., 2018; Strien et al., 2013).

Spatial survey bias is particularly problematic for countries, like Australia, where the vast majority of land mass is unpopulated and inaccessible, population density is low, and ecosystems are 100s or 1000s of kilometers from the nearest city. Consortium databases have been used in Australia to predict species distributions with climate change (Booth et al., 2012; González‐Orozco et al., 2014; Graham et al., 2019), including in freshwater environments (Bond et al., 2011; Graham et al., 2019; James et al., 2017). However, the underlying extent to which spatial survey effort bias exists in records, and the relative influence of this on species predictability has been given little attention (Boakes et al., 2010; Fithian et al., 2015; Piccolo et al., 2020). If survey bias means species distribution models fail to encapsulate a reasonable approximation of distribution patterns, then conservation and restoration planning will be misinformed, potentially leading to inappropriate management (Dormann, 2007; Guillera‐Arroita et al., 2015).

Without understanding survey effort in the data, species distribution modeling will continue to be challenging for managers, particularly when facing decisions to approve more anthropogenic impacts. Like the rest of the world, Australia faces a legacy of degraded freshwater ecosystems, despite a small population and a relatively short 200 years of urban, industrial, and agricultural development (Creighton et al., 2016). In the Great Barrier Reef (GBR) catchment, the loss and degradation of wetlands is also reducing the GBR’s resilience to pressures via ongoing pollutant runoff (Adame, 2019; Waterhouse et al., 2016) and reduced habitat availability for species with freshwater life stages (Adame, 2019; Arthington, 2015). This has sparked management goals seeking to maintain and improve the extent and condition of wetlands (State of Queensland, 2018). In addition to traditional pressures on wetlands (agriculture and urban development), there is increasing pressure to alter wetlands to capitalize on their carbon sequestration or water quality improvement services, which may have biodiversity consequences (Bell‐James & Lovelock, 2019; Stewart‐Sinclair et al., 2020; Waltham et al., 2019). Effectively managing wetland values within the GBR catchment has, in part, been constrained by a lack of understanding on biodiversity patterns or a clear set of agreed values and services, which may be constrained by survey bias (Boer, 2010; Burley et al., 2012; Shoo, 2014).

### 1.1 The changing environment

#### 1.1.1 Habitat loss and gain

Official wetland mapping across the Great Barrier Reef (GBR) catchment has been carried out by the Queensland Government since 2001 (Environmental Protection Agency, 2005), with the latest iteration released in 2017 that also includes estimates of preclear extent (Department of Environment & Science, 2019a). The mapping program uses a modified version of the Ramsar definition that excludes riparian zones above the saturation level and intermittently covered floodplains that do not meet the hydrophyte and soil criteria (Environmental Protection Agency, 2005). According to Wetland*Info*, across the GBR catchment, in 2017 approximately 90.5% of preclear estuarine areas (excluding open water), 96.1% of preclear lacustrine, 78.8% of preclear palustrine, and 83.5% of preclear riverine wetlands remained (Department of Environment & Science, 2019b). Between 2001 and 2017, there was a net loss of 7,688 ha across natural wetlands (i.e., excluding artificial/highly modified), with riverine wetlands accounting for 6,255 ha, estuarine salt flats and saltmarshes accounting for 605 ha, and coastal and subcoastal tree swamps (*Melaleuca* spp. and *Eucalypus* spp.) accounting for 569 ha and 537 ha on nonfloodplains and floodplains, respectively. Much of the decline attributed to clearing and drainage for urban and agricultural development. Artificial/highly modified wetlands (including dams, ring tanks, and irrigation channels), largely for irrigation water storage, accounted for the large majority of increased wetland, with approximately additional 21,546 ha in 2017 compared with 2001, representing a 15.2% increase. A substantial proportion of the artificial/highly modified wetlands was created through bunding (constructing a wall to exclude saltwater and retain freshwater) (Abbott, 2020), accounting for 8,299 ha of the increase (Department of Environment & Science, 2019b). These statistics do not include wetlands smaller than 1 ha as they are not mapped. The change in extent and composition of wetlands across the GBR catchment can be explored by subcatchment with further background information at the Queensland Government Wetland*Info* website (https://wetlandinfo.des.qld.gov.au/wetlands/facts‐maps/study‐area‐great‐barrier‐reef/).

#### 1.1.2 Climate change predictions

**FIGURE 1:**
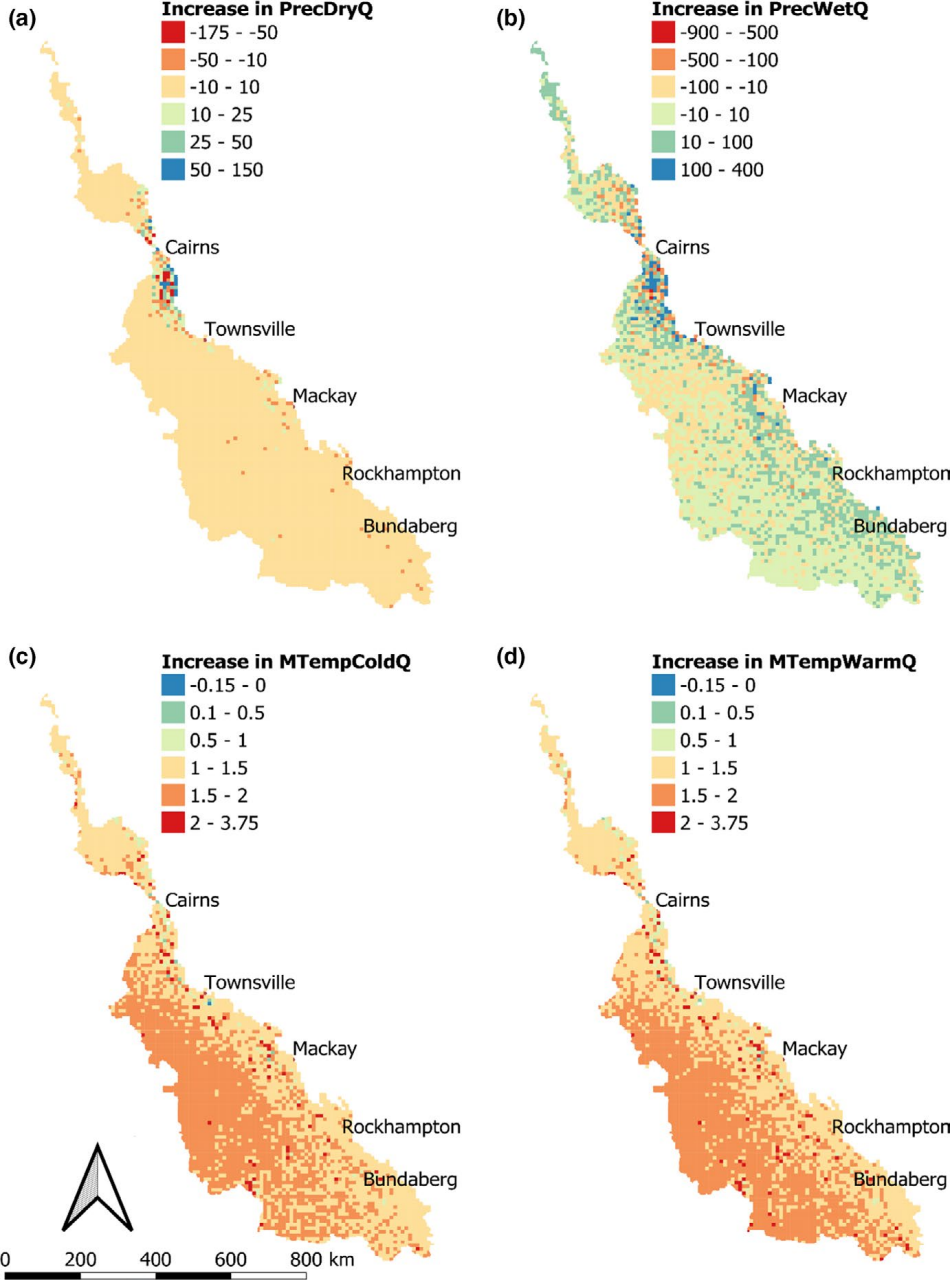
The difference in climate predictions for a high impact climate scenario (SSP 5–8.5) for 2081–2100 and baseline (1970–2000), sourced from Fick and Hijmans (2017), summarized as 10 × 10 km means. The future scenario predictions are the average of eight downscaled global climate models (BCC‐CSM2‐MR, CNRM‐CM6‐1, CNRM‐ESM2‐1, CanESM5, IPSL‐CM6A‐LR, MIROC‐ES2L, MIROC6 and MRI‐ESM2‐0; Fick & Hijmans, 2017). Change in mean precipitation for the driest and wettest quarters and change in mean temperature for the coldest and warmest quarters is presented in panels a–d, respectively

WorldClim 2 provides 19 climate metrics for baseline conditions and a range of future climate scenarios and models at 1 km resolutions (Fick & Hijmans, 2017). Globally, the cross‐validated correlations on baseline data were 0.86 for precipitation, 0.76 for wind speed, and ≥ 0.99 for temperature and humidity, though there is regional variation between models and parameters. Figure 1 compares the baseline (1970–2000) conditions for four climate variables with those predicted for 2081–2100 assuming a Shared Socioeconomic Pathway (SSP) 5–8.5 scenario. The SSP 5–8.5 scenario represents a high impact scenario with high fossil fuel consumption throughout the 21st century (Meinshausen et al., 2020). The future scenario predictions are the average of eight downscaled global climate models (BCC‐CSM2‐MR, CNRM‐CM6‐1, CNRM‐ESM2‐1, CanESM5, IPSL‐CM6A‐LR, MIROC‐ES2L, MIROC6 and MRI‐ESM2‐0; Fick & Hijmans, 2017). Overall, north of Cairns is predicted to become dryer, whereas southern parts, particularly south of Bundaberg, are predicted to become wetter. Inland areas across the entire catchment are also predicted to have wetter dry seasons (May to September). In terms of air temperature, the area spanning ~200 km around Cairns is predicted to have the largest changes, with the northern region predicted to be cooler and the southern region predicted to be warmer.

### 1.2 Study objectives

We used Australia's largest consortium biodiversity database (Belbin & Williams, 2016), the Atlas of Living Australia (ALA), to examine the influence of urban proximity on models of taxonomic richness and assemblage turnover, relative to habitat, landscape, and climate predictors, for five groups of wetland‐affiliated vertebrates (fish, amphibians, reptiles, birds, and mammals) within the GBR catchment. For clarity, vertebrates examined include all groups regardless of whether they are terrestrial or aquatic. If the relative influence of urban proximity is low, then the findings may be useful in predicting how wetland‐affiliated vertebrate distributions may respond to their changing environment. If the relative influence is high, then findings may still be useful if the model still has sufficient training data to learn patterns across other gradients. However, the results should be considered as having considerable uncertainty as identifying whether training data are sufficient to encapsulate other gradients is difficult. Furthermore, model variable selection may ignore other important predictors if they are highly correlated with urban proximity. Carrying out randomized surveys would be necessary to better encapsulate relationships and avoid spurious correlations. Careful interpretation, using existing knowledge, can also help identify whether detected patterns are likely plausible or spurious correlations.

## 2 METHODS

### 2.1 Environmental variables

We collated and summarized environmental metrics on climate, landscape position, and wetland habitat into 10 x 10 km grids from a lattice spanning the entire GBR catchment (*n* = 3,822 grids; Appendix S1). The baseline climate (1970–2000) estimates for 19 climate metrics were sourced from WorldClim 2 (Fick & Hijmans, 2017), with the mean then calculated for each grid. The extent (Ha) of each wetland habitat type within each grid was sourced from the Queensland wetland mapping (Department of Environment & Science, 2019a; Environmental Protection Agency, 2005). Within each grid, the number of wetland habitat types present (NumHabs) and the Simpson Diversity Index (Sim_div) was calculated using the presence and extent of each habitat type. The mean and majority of the Topographic Wetness Index (TWImean and TWImajorit, respectively) and Topographic Position Index (TPImean and TPImajorit, respectively) were also calculated from national landscape mapping (Gallant & Austin, 2012a, 2012b, 2012a, 2012b). The TWI estimates the relative wetness within a catchment, while the TPI is a measure of topographic position, classified into three classes corresponding to upper slopes, mid‐slopes, and lower slopes. For each grid cell, the Euclidian distance from the grid center to the center of the nearest city (Cairns, Townsville, Mackay, Rockhampton, and Bundaberg) was also calculated (Town_dis_km).

### 2.2 Vertebrate survey data

**FIGURE 2:**
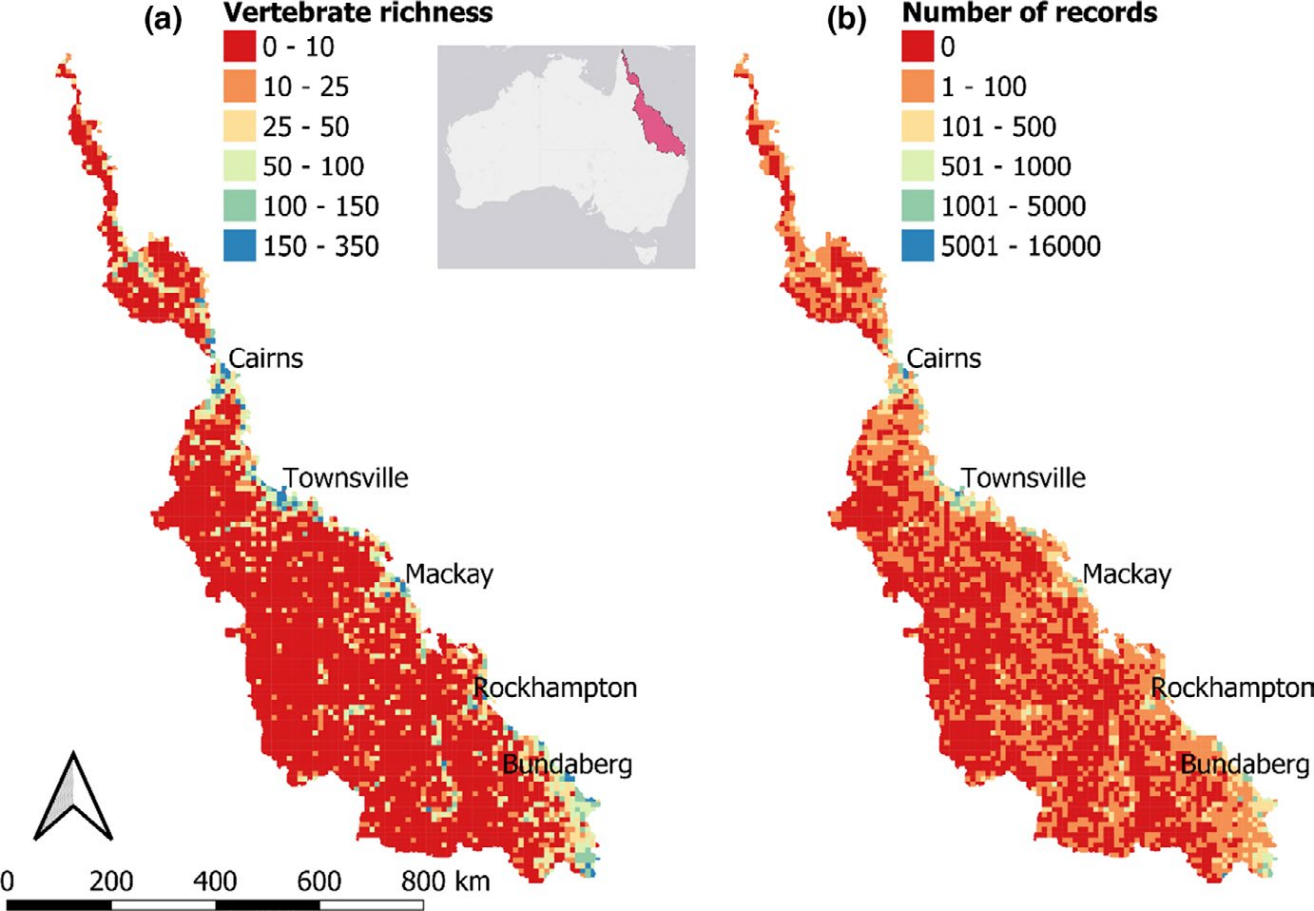
The (a) total number of wetland‐affiliated vertebrate taxa and (b) total number of Atlas of Living Australia (Belbin & Williams, 2016) survey records identified within 10 × 10 km grids across the Great Barrier Reef catchment (location in inset)

Vertebrate records from within the GBR catchment were extracted from the Atlas of Living Australia (ALA). ALA is a large database that collates sightings of animals from a wide range of organizations and contributors (Belbin & Williams, 2016). Any vertebrate record found within the Great Barrier Reef (GBR) catchment was extracted from the ALA database (Figure 2). This yielded 48,640 fish records, 56,415 amphibian records, 60,478 reptile records, 2,977,628 bird records, and 45,802 mammal records, collated from 351 datasets (dataset contributors in online species record data). Given the likely differences in survey method and intensity among observers, which could reduce the reliability of abundance data, this analysis only examined the presence of a species, rather than the abundance. It was assumed that lack of observations of the site indicated absence and wise use as a pseudoabsence, though this may not always be the case as the surveys were not exhaustive or systematic. Furthermore, not all species may be present at a site at one time, so the assemblages represent cumulative occupancy over time. Nonetheless, the ALA dataset represents the most comprehensive observation dataset over the entire spatial extent and is, therefore, the best data available for this analysis.

To avoid the inclusion of species with sporadic wetland use, species that were recorded fewer than 30 occasions within 100 m of a wetland were not considered to be wetland‐affiliated. A 100 m buffer was used to account for species that depend on riparian vegetation and birds that roost around the edge of wetlands. As this study was interested in native fauna richness and assemblages, exotic species were excluded.

**FIGURE 3:**
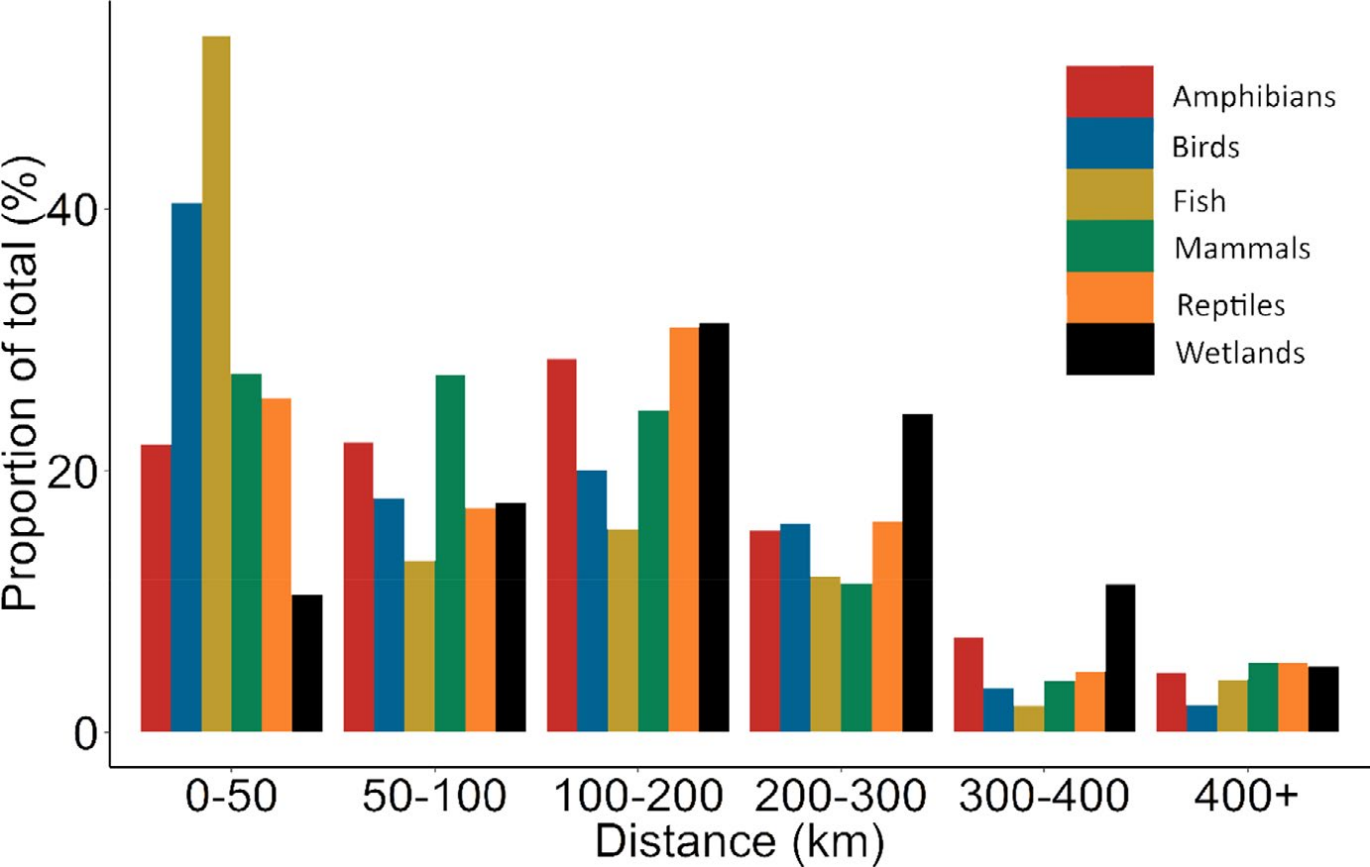
The proportion of Atlas of Living Australia (Belbin & Williams, 2016) survey records for wetland‐affiliated taxa in five vertebrate groups, and the proportion of wetlands (black), that are within given distances from the nearest city (km) across the Great Barrier Reef catchment. Vertebrate groups were amphibians (red); birds (blue); fish (gold); mammals (green), and reptiles (orange). Cities were Cairns, Townsville, Mackay, Rockhampton, and Bundaberg

Species occurrence records were summarized into a 10 x 10 km grids, spanning the GBR catchment area (*n* = 3,822 grids). For each grid, the richness of wetland‐affiliated taxa sighted within that grid was counted (see Figure 3 and Appendix S2–S7 for maps of taxonomic richness). Species were classified as being either a fish, amphibians, reptiles, birds, and mammals.

### 2.3 Exploration of taxonomic richness patterns

For each of the five vertebrate groups, boosted regression tree (BRT) modeling was used to explore potential relationships between the taxon richness and the environmental variables estimated for each grid (see Appendix S8–S17). BRTs are a powerful and standard machine‐learning technique that create many regression trees that are combined in a forward, stepwise fashion to improve predictor performance (Elith et al., 2008; Pichler et al., 2020). Regression trees relate a response variable to predictors via recursive binary splits. BRTs are capable of fitting complex interactions, nonlinear predictors, and handling non‐normal error terms and missing values. The method combines the strengths of two algorithms: regression trees (models that relate a response to their predictors by recursive binary splits) and boosting (an adaptive method for combining many simple models to give improved predictive performance). Trees are added and assessed sequentially until the holdout deviance is minimized, which reduces the probability of overfitting (Elith et al., 2008; Pichler et al., 2020). The relative influence of each factor was calculated following Friedman and Meulman (2003), and was the average number of times a variable was selected for a split, weighted by the squared improvement to the model at each split, and then scaled to ensure all variable importance scores add to 100 (Elith et al., 2008; Friedman & Meulman, 2003). All five BRTs were run with a tree complexity of 7, learning rate of 0.01, and cross‐validated using a bag fraction of 0.2, using the Dismo package (Hijmans et al., 2012) in R (R Core Team, 2016).

### 2.4 Exploration of assemblage patterns

Gradient forests are an extension of the random forest modeling approach and assess how the compositional turnover of ecological assemblages changes over gradients (Ellis et al., 2012). Random forests are a collection of regression trees, whereby each tree is fitted to a bootstrapped sample (with replacement) and then validated on the out‐of‐bag sample (Breiman, 2001). Random forest predictions are the average of the predictions of each tree. Regression trees, and consequently random forests, work by partitioning observations at splits of predictors that minimize the sum of squares error. They have a high level of flexibility, and can handle nonlinear relationships and complex interactions (Cutler et al., 2007; Ellis et al., 2012; Hastie et al., 2009). The R package *extendedForest* (Ellis, 2019a) not only computes the Breiman and Cutler's random forests, but also records the raw importance, and can calculate the conditional (instead of marginal) permutation importance of correlated predictors for a given correlation, following Strobl et al. (2008) and Ellis (2012). The overall fit for each species was assessed using a statistic analogous to r^2^, in which r^2^ = 1 – OOB (out of bag) misclassification rate/base error rate (BE), with BE = 2p(1–p) and p is species prevalence (Ellis et al., 2012).

In the gradient forest approach, the *extendedForest* package (Ellis, 2019a) is used to grow univariate random forests that predict the probability of occurrence for each species from environmental predictors. Species turnover across an environmental gradient is indicated by the amount of change across neighboring partitions of a given split. The *gradientForest* package (Ellis, 2019b) calculates assemblage compositional turnover by aggregating the change at each split for the entire assemblage (when cross‐validated *R* ^2^ > 0), with each species weighted by its goodness of fit and predictor importance (see Ellis (2012) for more details).

For each taxonomic group, gradient forests were used to explore potential relationships between assemblage turnover and the environmental variables estimated for each grid (see Appendix S18–S27). Gradient forests were modeled using the *extendedForest* and *gradientForest* packages (Ellis, 2019a, 2019b, 2019a, 2019b) in R (R Core Team, 2016), with 500 bootstrapped trees generated for each random forest.

## 3 RESULTS

### 3.1 Taxonomic richness patterns

All BRT explorations of taxonomic richness performed moderately well, with bird richness predictions outperforming the other groups (birds CV *r* ^2^ = 0.64, others CV *r* ^2^ = 0.48–0.5; Table 1; Appendix S8–S17). The BRT explorations of each vertebrate group consistently showed that the distance from the nearest city was the most influential factor (or second most for fish) that predicted taxonomic richness. For all groups, diversity was predicted to reduce with increasing distance, with little change beyond 100 km from a city. In addition to city proximity, wetland habitat area was also highly influential in predicting taxonomic richness, which increases with increasing area. Climate variables were less prevalent among the most influential factors; however, high rainfall in the driest quarter was associated with greater richness for amphibians and mammals, and high maximum temperatures associated with lower mammal richness.

**TABLE 1:** The six most influential predictors, and the test statistics, from five boosted regression tree (BRT) models predicting the species richness, and five gradient forest (GF) models predicting the assemblage turnover, of five vertebrate groups across the Great Barrier Reef watersheds

	Factor/statistic	Fish	Amphibians	Reptiles	Birds	Mammals
BRT of taxonomic richness	Factor 1	Hab_60	Town_dis_km	Town_dis_km	Town_dis_km	Town_dis_km
	Factor 2	Town_dis_km	PrecDryQ	Hab_60	Hab_4c	MaxTWarmMo
	Factor 3	Hab_32	Hab_50	All_wetlan	Hab_40	Hab_4a
	Factor 4	Hab_30	Hab_40	Hab_40	Hab_30	Hab_2a
	Factor 5	Hab_50	Sim_div	Hab_4a	All_wetland	PrecDryQ
	Factor 6	All_wetlan	All_wetlan	Sim_div	Sim_div	All_wetlan
	CV correlation (*r* ^2^)	0.54	0.50	0.50	0.69	0.49
	CV correlation SE	0.031	0.018	0.025	0.008	0.034
GF of assemblage turnover	Factor 1	Sim_div	Town_dis_km	Town_dis_km	Town_dis_km	Town_dis_km
	Factor 2	Hab_32	PrecDryQ	PrecColdQ	TempSD	All_wetlan
	Factor 3	Hab_31	PrecColdQ	TempSD	MTempColdQ	Isothermal
	Factor 4	MaxTWarmMo	MTempColdQ	MTempColdQ	Hab_31	PrecDryQ
	Factor 5	Hab_60	TempSD	PrecDryQ	Hab_40	PrecColdQ
	Factor 6	PrecColdQ	AnnMeanTemp	All_wetlan	TempRange	TempSD
	Average *r* ^2^	0.64	0.44	0.50	0.47	0.48
	*SD* of all *r* ^2^	0.19	0.09	0.09	0.11	0.08
	Lowest *r* ^2^	0.11	0.25	0.37	0.23	0.32
	Highest *r* ^2^	0.95	0.62	0.82	0.86	0.79

Abbreviations: CV, cross‐validated; SE, standard error.

### 3.2 Group assemblage patterns

For each of the five vertebrate groups, the random forests predicting each species had variable performance, ranging from excellent to poor (Table 1; Appendix S18–S27). For all vertebrate groups, except fish, the distance from the nearest city was the most influential factor (those with the highest overall weighted r^2^ importance) in predicting assemblage turnover. The greatest turnover typically occurred within the first 100 km from a city. The precipitation occurring in the driest/coldest quarter was also a highly influential factor predicting assemblage turnover for all groups, except birds. The variability in temperature (TempSD) and the temperature in the coldest quarter were also influential for the turnover of all groups, except fish turnover, which was influenced by the warmest temperatures.

## 4 DISCUSSION

City proximity was consistently the most influential factor predicting the richness and assemblages of the vertebrate groups, except fish, despite there being an array of wetland habitat, climate, and landscape variables as potential predictors. The greatest change in richness or assemblage turnover was predicted to occur within the first 100 km of a city, possibly reflecting the willingness of animal watching hobbyists (who generally contributed the most ALA records) to travel for their recreation. Consequently, the data were skewed toward cities, rather than representative of wetland distribution, which means that data for wetlands that are more distant from urban centers were not available, which severely limits the ability to run species distribution modeling. It is not uncommon for citizen science programs, outside a structured or randomized assessment program, to yield greater species detection rates closer to home (Sicacha‐Parada et al., 2020; Tulloch & Szabo, 2012). Not assessed here is also the potential impact of land accessibility and the potential influence of small towns between cities, which can both be potential sources of noise. While citizen science records can be useful, it is not always the case (Steen et al., 2019), and systematic surveys and long‐term monitoring are still needed to develop and validate species distribution models, and divulge any spurious correlations among predictors (Sinclair et al., 2010).

It may be reasoned that the influence of urban proximity could be a spurious correlation as human settlements are traditionally proximal to freshwater or wet climates (Duranton, 1999; Small & Nicholls, 2003). It may also be reasoned that some species may benefit from the environments within urban centers (e.g., migrating birds for the artificial ponds). However, in the GBR catchment the distance to the nearest city was poorly correlated with other predictors, including wetland habitat extent (which included artificial habitats in urban environments) and rainfall. Therefore, the minimization of variable importance for other predictors (which can occur with tree‐based models), due to predictor correlation with urban proximity, is unlikely. Furthermore, despite city proximity being highly influential factor in all models, relationships observed with other predictors may still provide insight into relationships between wetland‐affiliated vertebrates and other predictors, such as habitat extent and climate. However, it is cautioned that the results would be uncertain if models did not have sufficient training data to encapsulate alternative patterns or if variables are highly correlated.

Fish were the only group assessed where urban proximity was not the most influential factor predicting taxonomic richness and assemblage turnover, though still highly influential. The richness of fishes was predicted to increase with increasing marine, estuarine, and riverine habitat. Estuarine habitat extent and wetland habitat diversity (Simpson's) were the most influential factors affecting fish turnover. Several plausible, and not necessarily mutually exclusive, explanations exist: (a) barriers prevent upstream colonization; (b) the presence of invasive fish was not included as a predictor; (c) coastal habitats support many niches; or (d) evolutionary legacies from marine ancestors. Fish barriers are prevalent across many parts of the GBR catchment and may be preventing upstream colonization (Kroon & Phillips, 2016). This study did not include fish barriers as an environmental variable. Further work is required to map these barriers and examine their impact on upstream fish assemblages (Waltham et al., 2019). Invasive fish, such as Tilapia, are found in parts of the catchment, particularly near cities as aquarium fish are released into nearby waterways. It is plausible that their presence is excluding other fish. Estuarine and coastal marine habitats, such as mangrove and seagrass, are highly productive and diverse habitats, potentially allowing a large range of species to persist. Furthermore, unlike in many other parts of the world, Queensland (and Australia in general) has relatively few primary freshwater fish (Pusey et al., 2004; 2017), the majority being secondary freshwater species that have evolved from marine ancestors (Pusey et al., 2004; Williams & Allen, 1987). This is hypothesized to be driven by the extreme seasonal variation facing many freshwater ecosystems in Australia, particularly across the GBR catchment where they are highly disturbed by floods in the wet season and drought in the dry season, with marine habitats providing a stable source of fish for freshwater habitats (Pusey et al., 2004; Williams & Allen, 1987). The legacy of disturbance extremes and marine ancestors may also explain why estuarine and coastal habitat is highly influential for fish taxonomic richness and assemblages.

Amphibian taxonomic richness and assemblage turnover were predicted to increase with increasing dry season rainfall, and richness increase with greater riverine and artificial wetland habitat extent. The species predicted to be most influenced by dry season rainfall were the Pearson's green tree frog, Giant barred frog, Bridled frog, Long‐thumbed frog, and the New Holland frog. During the dry season, many wetlands either desiccate or retreat substantially. As a result, species dependent on wetlands may need to migrate to permanently wet areas, and then potentially face high competition with other species present. Higher rainfall during the dry season may alleviate competition and movement pressures, and allow species that do not move large distances to persist (Laurance, 1996; Rowley & Alford, 2007; Williams & Hero, 2001). As with other vertebrate groups, alterations in dry season rainfall predicted to occur in the Russell and Johnstone river catchments, 50–100 km south of Cairns, may exacerbate impacts associated with the loss of riverine habitats (including riparian) within the area. Restoring riverine habitat (including riparian) within these river catchments, and managing irrigation takes during the dry season, may help alleviate the potential stress arising from the disproportionately high rainfall reductions during the dry season in the catchments.

For reptiles, aside from urban proximity, richness was highly influenced by the extent of marine habitat and total wetland habitat, predicted to increase until ~50% and ~60% grid coverage, respectively. Although the richness models of reptiles were largely influenced by habitat extent, the gradient forest models suggest climatic variables were more important for assemblage turnover. In particular, reptile assemblage turnover is largely predicted to be influenced by the rainfall and temperature during the coldest quarter. While temperature was similarly influential across the entire gradient, the precipitation during the coldest quarter (mid‐dry season) was most influential up to 300 mm and then began plateauing until ~600 mm. The drying predicted to occur with extreme climate change may be most impactful on altering reptile assemblages in the Johnstone and Russell river catchments, 50–100 km south of Cairns.

Bird taxonomic richness was predicted to increase with increasing wetland extent, primarily with increases in coastal/subcoastal floodplain grass, sedge, and herb swamps and in artificial/highly modified wetlands. Assemblages were predicted to change gradually over gradients of the mean temperature in the coldest quarter (dry season) and temperature variability. The extent of coastal/subcoastal floodplain grass, sedge, and herb swamps has changed very little between 2001 and 2017, while the extent of artificial/highly modified wetlands has increased substantially; this may have yielded positive benefits for bird taxonomic richness. Much of the artificial/highly modified wetlands have been created for ponded pasture to provide cattle with forage during the late dry season. Whiles these wetlands are unnatural, they provide large areas of shallow fresh water with herbaceous plants on coastal floodplains that could provide similar support to the natural coastal/subcoastal floodplain grass, sedge, and herb swamps. Given the increasing interest within the GBR catchment to convert ponded pastures to mangrove for carbon abatement (Kelleway et al., 2017), further effort should be taken to survey bird assemblages supported by ponded pastures to ensure the inevitable value trade‐offs are well informed (Bell‐James & Lovelock, 2019; Stewart‐Sinclair et al., 2020; Waltham et al., 2019).

The taxonomic richness of mammals is predicted to reduce with warmer maximum temperatures (MaxTWarmMo) and increase with greater coastal/subcoastal tree swamps (Melaleuca and Eucalypt). The turnover of mammal assemblages was predicted to be influenced by wetland extent (entire gradient), isothermality (largely 45–50), and low precipitation during the driest quarter (mid‐dry season; <200 mm). The richness of mammals may have been impacted in the coastal areas surrounding Bundaberg and the region ~200–500 km west and north‐west of Rockhampton, where large losses of coastal/subcoastal tree swamps occurred. While extreme climate change is not predicted to affect isothermality greatly across the GBR catchment, reduced dry season rainfall in the Russell and Johnstone catchments (as predicted) may alter the local mammal assemblages.

In summary, over the past few decades the GBR catchment has lost large areas of coastal/subcoastal tree swamp (Melaleuca and Eucalypt), which could be affecting mammal assemblages. However, this has been partially offset by increases in the extent of artificial/highly modified wetlands that may be benefiting bird assemblages. Rainfall during the driest quarter was frequently one of the most influential climatic factors affecting wetland‐affiliated vertebrates, particularly reptiles, mammals, and amphibians. If extreme climate change predictions are realized, then the Russell and Johnstone river catchments, south of Cairns (predicted to have the largest reductions in rainfall), may experience substantial changes in the wetland‐affiliated vertebrates assemblages. Restoring wetland habitats for the most affected species within these catchments may improve their resilience to climate change. Despite these findings, the survey data used were heavily biased toward cities, and this was highly influential on all models of richness and assemblage turnover. Having models informed by highly biased data is problematic for conservation and restoration planning as it makes it incredibly difficult to then predict plausible outcomes from enacting various management levers, which may lead to inappropriate or less than desired outcomes. We advocate here that similarly high levels of bias would be observed elsewhere and consider the influence requires greater attention when modeling species distributions in future or interpreting those made in the past. We urge managers to carry out a systematic and randomized survey of vertebrates in wetlands (including artificial) across the GBR catchment to ensure conservation management can effectively and efficiently manage wildlife values with a changing environment.

## CONFLICT OF INTEREST

No conflicts to declare.

## AUTHOR CONTRIBUTION

**Adam Canning:** Conceptualization (lead); Formal analysis (lead); Investigation (lead); Methodology (lead); Visualization (lead); Writing‐original draft (lead); Writing‐review & editing (lead). **Nathan J. Waltham:** Conceptualization (supporting); Funding acquisition (lead); Investigation (supporting); Methodology (supporting); Project administration (lead); Writing‐review & editing (supporting).

## ETHICS STATEMENT

No ethics approval was required for this study.

### Open Research Badges

This article has earned an Open Data Badge for making publicly available the digitally‐shareable data necessary to reproduce the reported results. The data is available at Species record data available from https://doi.ala.org.au/doi/10.26197/5eccb9b944924 (mammals); https://doi.ala.org.au/doi/10.26197/5eccb9c070853 (reptiles); https://doi.ala.org.au/doi/10.26197/5eccba5b63190 (fish); https://doi.ala.org.au/doi/10.26197/5eccd2b233c9e (birds) and https://doi.ala.org.au/doi/10.26197/5ecdbb0e785e7 (amphibians). All wetland mapping data (version 5) is available from http://qldspatial.information.qld.gov.au/catalogue/custom/detail.page?fid=%7B986BE78D‐FA59‐4A9E‐92C5‐8626E50CF3A8%7D Model fit and variable importance statistics from random forests used in gradient forest modelling for all taxa examined is available from https://doi.org/10.5061/dryad.8931zcrq4.

## Supporting information

Appendix S1‐S27

## Data Availability

Species record data available from https://doi.ala.org.au/doi/10.26197/5eccb9b944924 (mammals); https://doi.ala.org.au/doi/10.26197/5eccb9c070853 (reptiles); https://doi.ala.org.au/doi/10.26197/5eccba5b63190 (fish); https://doi.ala.org.au/doi/10.26197/5eccd2b233c9e (birds); and https://doi.ala.org.au/doi/10.26197/5ecdbb0e785e7 (amphibians). All wetland mapping data (version 5) are available from http://qldspatial.information.qld.gov.au/catalogue/custom/detail.page?fid=%7B986BE78D‐FA59‐4A9E‐92C5‐8626E50CF3A8%7D. Model fit and variable importance statistics from random forests used in gradient forest modeling for all taxa examined are available from https://doi.org/10.5061/dryad.8931zcrq4.
