# Supplementary material for: Ecological impact assessment of climate change and habitat loss on wetland vertebrate assemblages of the Great Barrier Reef catchment and the influence of survey bias
Source: Ecol Evol. 2021 Mar 24;11(10):5244–54. doi: 10.1002/ece3.7412 (PMC8131784; doi:10.1002/ece3.7412)
Supplement: Supplementary file 1 — Appendix S1‐S27 [file ECE3-11-5244-s001.docx]

**Supporting Information**

**Appendix S1**

| **Appendix S1.** Description of the climate variables sourced from Fick and Hijmans (2017), landscape variables sourced from Gallants and Austin (2012b, 2012a), and wetland habitat extent and diversity variables from the Queensland wetland mapping (version 5) (Environmental Protection Agency 2005, Department of Environment and Science 2019a), calculated for each 10 x 10 km grid squares within a lattice covering the Great Barrier Reef catchment, Australia. | | |
| --- | --- | --- |
| Variable group | Variable code | Description |
| Climate variables | PrecColdQ | Precipitation of Coldest Quarter |
|  | PrecWarmQ | Precipitation of Warmest Quarter |
|  | PrecDryQ | Precipitation of Driest Quarter |
|  | PrecWetQ | Precipitation of Wettest Quarter |
|  | PrecCOV | Precipitation Seasonality (Coefficient of Variation) |
|  | PrecDryMonth | Precipitation of Driest Month |
|  | PrecWetMonth | Precipitation of Wettest Month |
|  | AnnPrec | Annual Precipitation |
|  | MTempColdQ | Mean Temperature of Coldest Quarter |
|  | MTempWarmQ | Mean Temperature of Warmest Quarter |
|  | MTempDryQ | Mean Temperature of Driest Quarter |
|  | MTempWetQ | Mean Temperature of Wettest Quarter |
|  | TempRange | Temperature Annual Range (MaxTWarmMonth-MinTColdMonth) |
|  | MinTColdMonth | Min Temperature of Coldest Month |
|  | MaxTWarmMonth | Max Temperature of Warmest Month |
|  | TempSD | Temperature Seasonality (standard deviation ×100) |
|  | Isothermality | Isothermality (MeanDiurnTRange/TempRange) (×100) |
|  | MeanDiurnTRange | Mean Diurnal Range (Mean of monthly (max temp - min temp)) |
|  | AnnMeanTemp | Annual Mean Temperature |
|  | TWImean | Topographic Wetness Index (mean) |
|  | TWImajorit | Topographic Wetness Index (majority) |
|  | TPImean | Topographic Position Index (mean) |
|  | TPImajorit | Topographic Position Index (majority) |
|  | All_wetland | The total area (Ha) of wetland habitats |
|  | Sim_div | The Simpson’s Diversity Index of wetland habitat types (area weighted) |
| Wetland habitat | 11c | Arid/ Semi-arid floodplain grass, sedge, herb swamps |
|  | 11a | Arid/ Semi-arid floodplain tree swamps |
|  | 15 | Arid/ Semi-arid fresh floodplain lakes |
|  | 16a | Arid/ Semi-arid fresh non-floodplain lakes |
|  | 13 | Arid/ Semi-arid non-floodplain (spring) swamps |
|  | 12c | Arid/ Semi-arid non-floodplain grass, sedge, herb swamps |
|  | 12a | Arid/ Semi-arid non-floodplain tree swamps |
|  | 10 | Arid/ Semi-arid saline swamps |
|  | 40 | Artificial/ highly modified wetlands (dams, ring tanks, and irrigation channels) |
|  |  | *Created by bunding (Hab_40_bund)* |
|  |  | *Created by damming (Hab_40_dam)* |
|  | 4c | Coastal/ Sub-coastal floodplain grass, sedge and herb swamps |
|  | 6 | Coastal/ Sub-coastal floodplain lakes |
|  | 4a | Coastal/ Sub-coastal floodplain tree swamps (Melaleuca and Eucalypt) |
|  | 4b | Coastal/ Sub-Coastal floodplain wet heath swamps |
|  | 3 | Coastal/ Sub-Coastal non-floodplain (spring) swamps |
|  | 2c | Coastal/ Sub-coastal non-floodplain grass, sedge and herb swamps |
|  | 7 | Coastal/ Sub-coastal non-floodplain rock lakes |
|  | 8 | Coastal/ Sub-coastal non-floodplain sand lakes |
|  | 8a | Coastal/ Sub-coastal non-floodplain sand lakes (Window) |
|  | 9 | Coastal/ Sub-coastal non-floodplain soil lakes |
|  | 2a | Coastal/ Sub-Coastal non-floodplain tree swamps (Melaleuca and Eucalypt) |
|  | 2b | Coastal/ Sub-Coastal non-floodplain wet heath swamps |
|  | 1 | Coastal/ Sub-Coastal saline swamps |
|  | 5 | Coastal/ Sub-Coastal tree swamps (palm) |
|  | 30 | Estuarine - Mangroves and related tree communities |
|  | 31 | Estuarine - salt flats and saltmarshes |
|  | 32 | Estuarine - water |
|  | 60 | Marine |
|  | 50 | Riverine |
| Landscape | TWImean | Topographic Wetness Index (mean) |
|  | TWImajorit | Topographic Wetness Index (majority) |
|  | TPImean | Topographic Position Index (mean) |
|  | TPImajorit | Topographic Position Index (majority) |
|  | All_wetland | The total area (Ha) of wetland habitats |
|  | Sim_div | The Simpson’s Diversity Index of wetland habitat types (area weighted) |
|  | Town_dis_km | The Euclidian distance (in 50 km intervals) to the nearest city |

**Appendix S2**

**
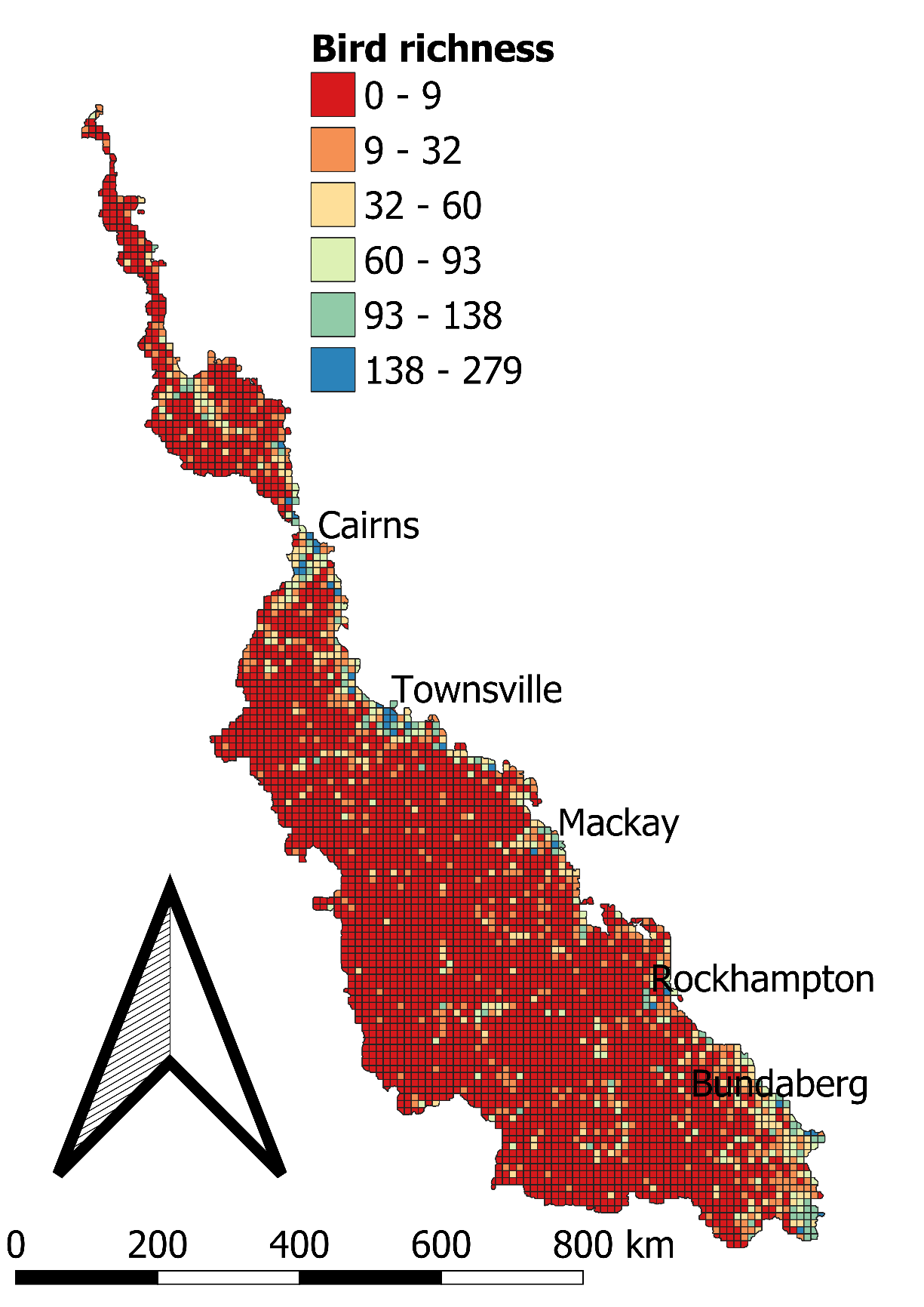
**

**Appendix S2.** The total number of water-affiliated bird species detected within 10 x 10 km grids across the Great Barrier Reef watershed as logged on the Atlas of Living Australia (doi.org/10.26197/5eccd2b233c9e).

**Appendix S3**


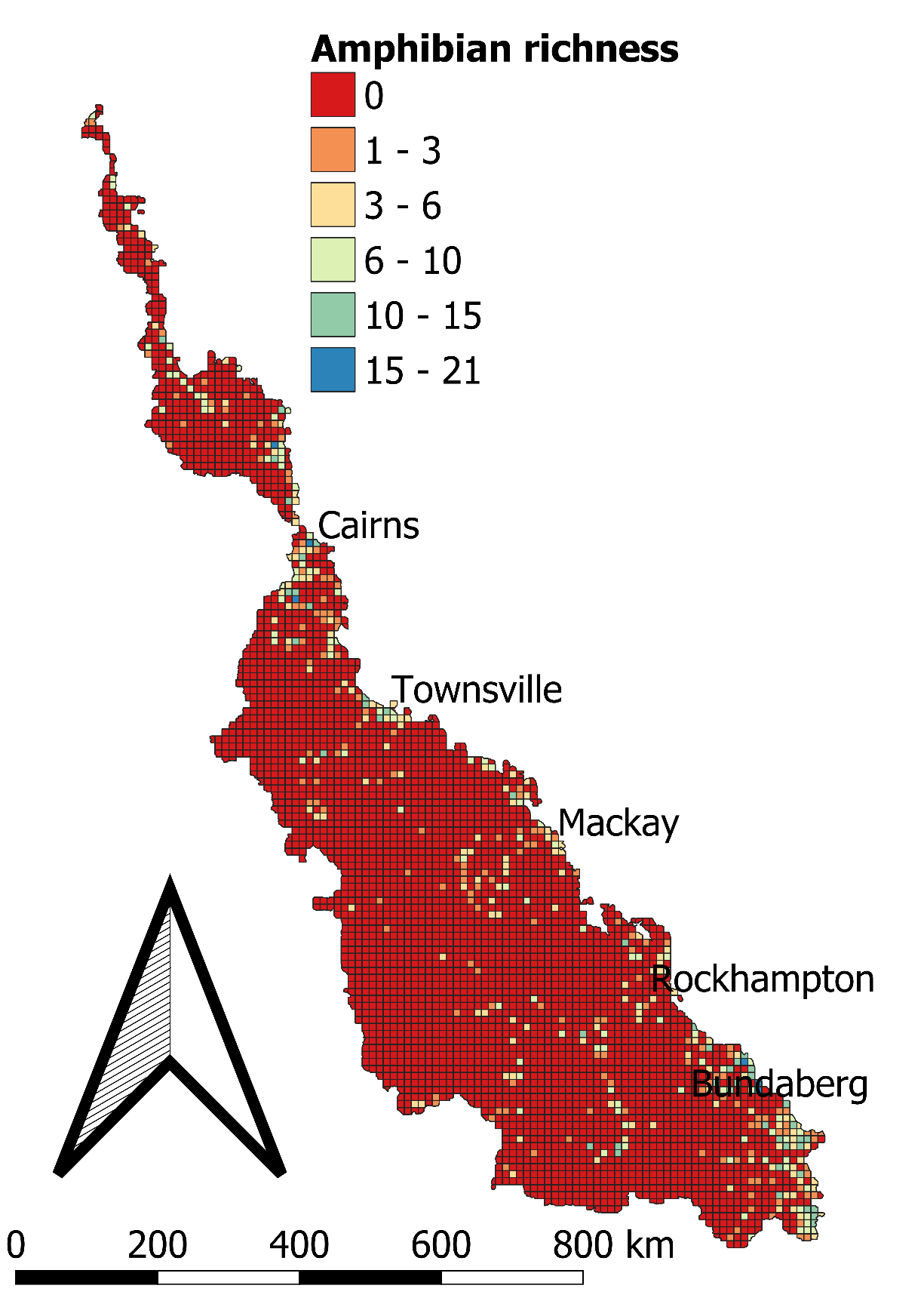


**Appendix S3.** The total number of amphibian species detected within 10 x 10 km grids across the Great Barrier Reef watershed as logged on the Atlas of Living Australia (doi.org/10.26197/5ecdbb0e785e7).

**Appendix S4.**


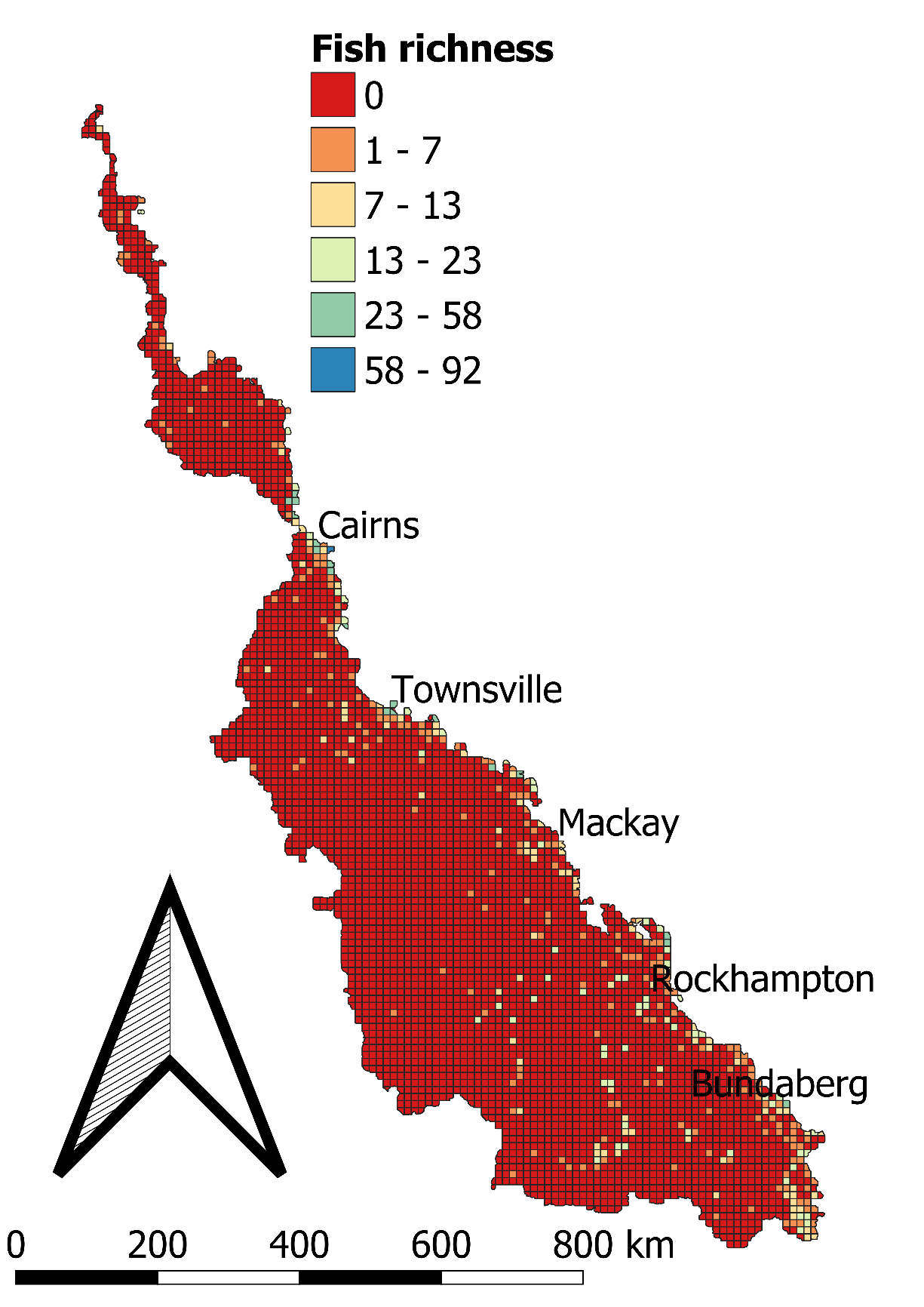


**Appendix S4.** The total number of fish species detected within 10 x 10 km grids across the Great Barrier Reef watershed as logged on the Atlas of Living Australia (doi.org/10.26197/5eccba5b63190).

**Appendix S5**


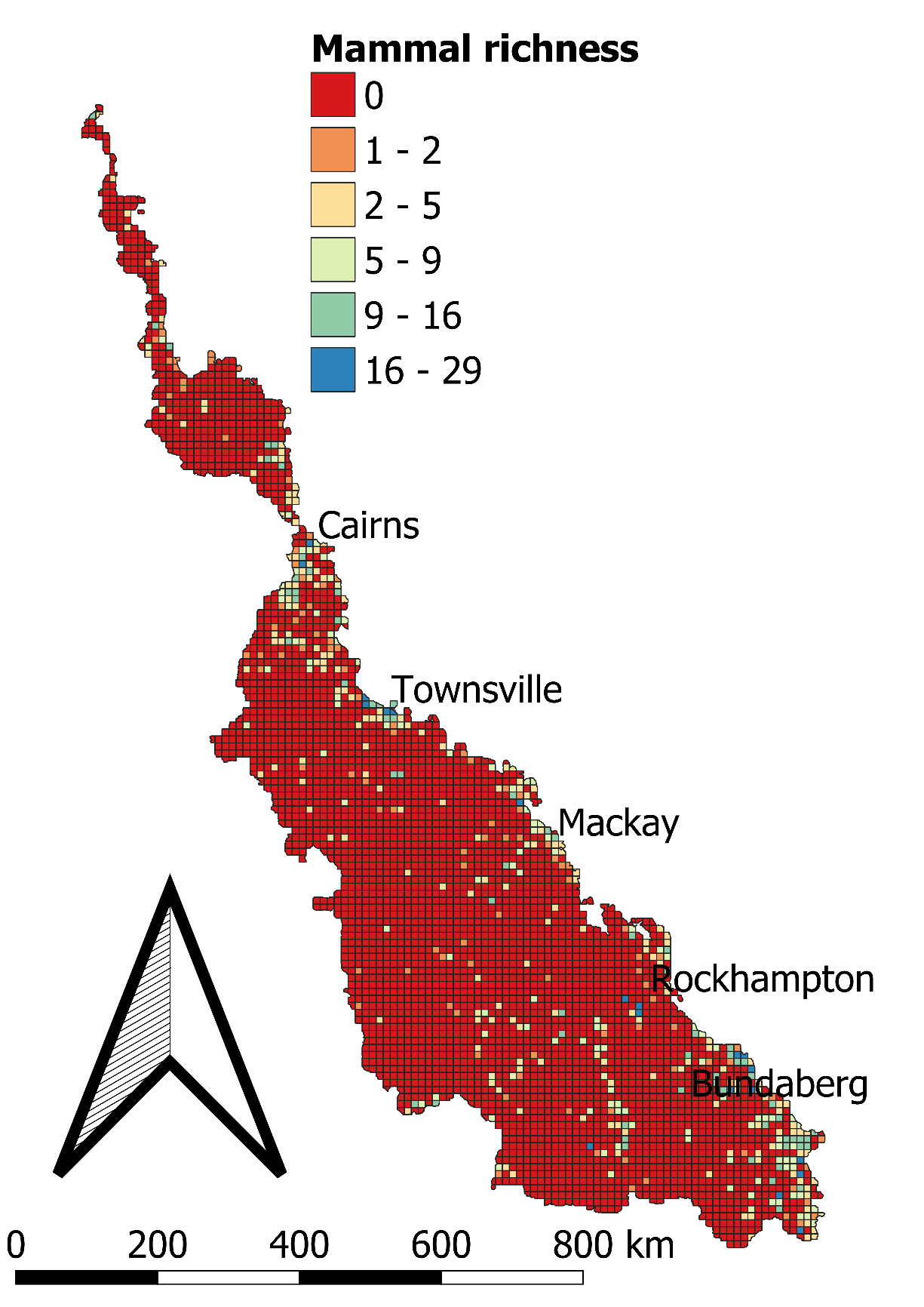


**Appendix S5.** The total number of water-affiliated mammal species detected within 10 x 10 km grids across the Great Barrier Reef watershed as logged on the Atlas of Living Australia (doi.org/10.26197/5eccb9b944924).

**Appendix S6.**


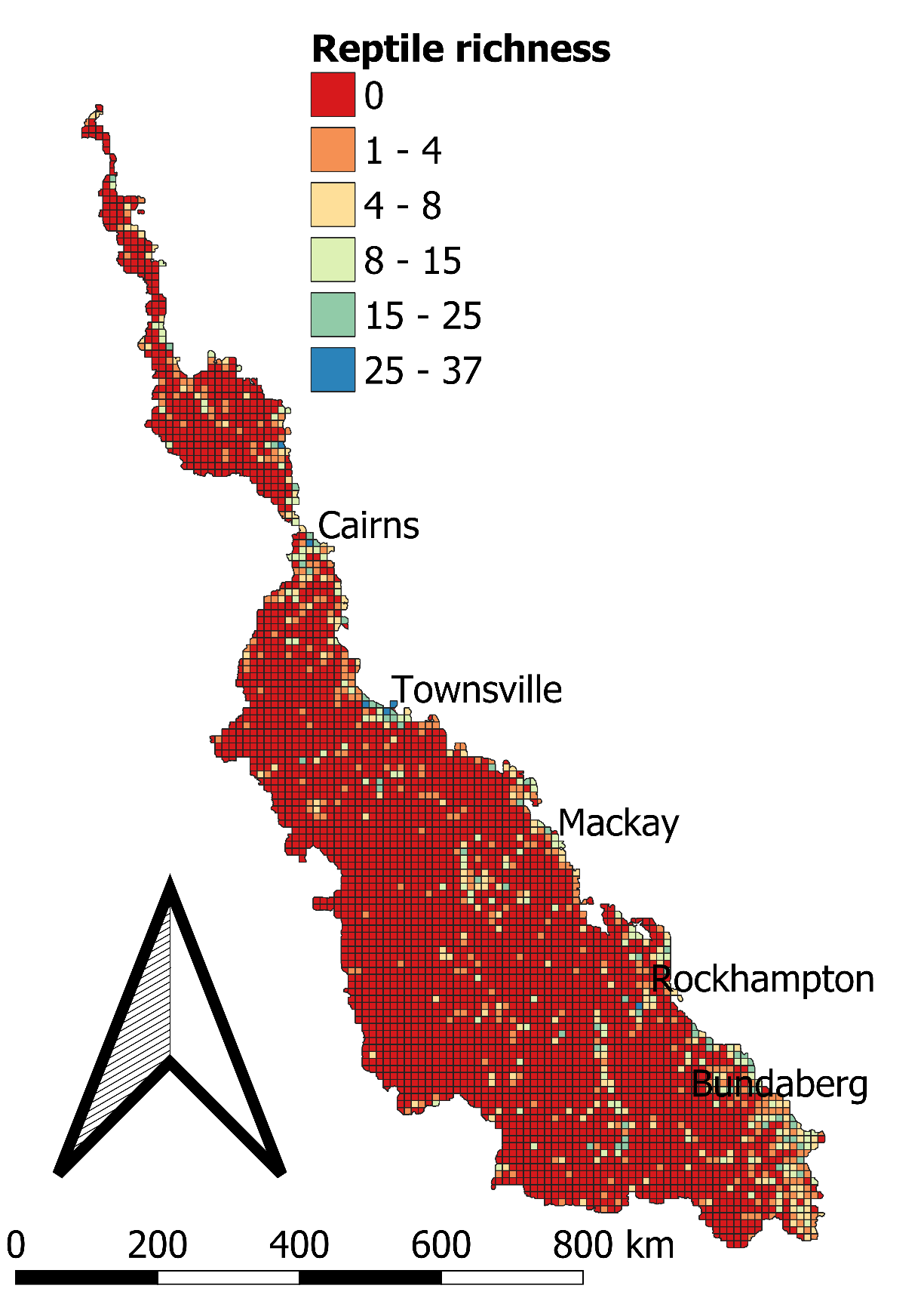


**Appendix S6.** The total number of water-affiliated reptile species detected within 10 x 10 km grids across the Great Barrier Reef watershed as logged on the Atlas of Living Australia (doi.org/10.26197/5eccb9c070853).


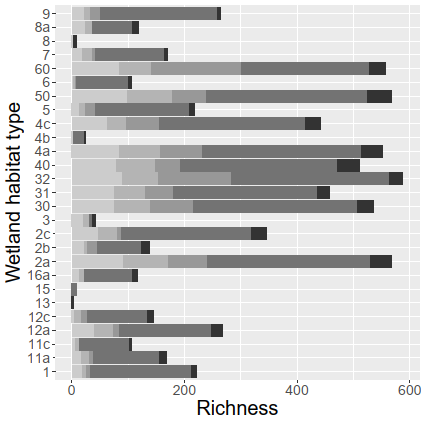
**Appendix S7**

**Appendix S7.** The total number of water-affiliated species within groups of amphibians, birds, fishes, mammals and reptiles (black to light grey respectively) detected within different wetland habitat types across the Great Barrier Reef watershed.

**Appendix S8**

**Appendix S8.** The relative influence of each the variables used in the BRT to predict amphibian richness.

**Appendix S9**

**Appendix S9.** The marginal effects of the top six most influential variables used in the BRT to predict amphibian richness.

**Appendix S10**

**Appendix S10.** The relative influence of each the variables used in the BRT to predict fish richness.

**Appendix S11**

**Appendix S11.** The marginal effects of the top six most influential variables used in the BRT to predict fish richness.

**Appendix S12**

**Appendix S12.** The relative influence of each the variables used in the BRT to predict mammal richness.

**Appendix S13**

**Appendix S13.** The marginal effects of the top six most influential variables used in the BRT to predict mammal richness.

**Appendix S14**

**Appendix S14.** The relative influence of each the variables used in the BRT to predict reptile richness.

**Appendix S15**

**Appendix S15.** The marginal effects of the top six most influential variables used in the BRT to predict reptile richness.

**Appendix S16**

**Appendix S16.** The relative influence of each the variables used in the BRT to predict bird richness.

**Appendix S17**

**Appendix S17.** The marginal effects of the top six most influential variables used in the BRT to predict bird richness.

**Appendix S18**

**Appendix S18.** The overall weighted R^2^ importance of each variable predicting a gradient forest model of amphibians across the Great Barrier Reef watershed.

**Appendix S19**

**Appendix S19.** The overall weighted R^2^ importance of each variable predicting a gradient forest model of reptiles across the Great Barrier Reef watershed.

**Appendix S20**

**Appendix S20.** The overall weighted R^2^ importance of each variable predicting a gradient forest model of mammals across the Great Barrier Reef watershed.

**Appendix S21**

**Appendix S21.** The overall weighted R^2^ importance of each variable predicting a gradient forest model of fishes across the Great Barrier Reef watershed.

**Appendix S22**

**Appendix S22.** The overall weighted R^2^ importance of each variable predicting a gradient forest model of birds across the Great Barrier Reef watershed.

**Appendix S23**

**Appendix S23.** The cumulative importance of the four most influential predictors in predicting the overall turnover of reptile assemblages (solid line) and the five most responsive species for each variable across the Great Barrier Reef watershed. The file ‘Species_fits_and_variable_importance.xlsx’ indicates the species represented by each the remaining five lines.

**Appendix S24**

**Appendix S24.** The cumulative importance of the four most influential predictors in predicting the overall turnover of fish assemblages (solid line) and the five most responsive species for each variable across the Great Barrier Reef watershed. The file ‘Species_fits_and_variable_importance.xlsx’ indicates the species represented by each the remaining five lines.

**Appendix S25**

**Appendix S25.** The cumulative importance of the four most influential predictors in predicting the overall turnover of mammal assemblages (solid line) and the five most responsive species for each variable across the Great Barrier Reef watershed. The file ‘Species_fits_and_variable_importance.xlsx’ indicates the species represented by each the remaining five lines.

**Appendix S26**

**Appendix S26.** The cumulative importance of the four most influential predictors in predicting the overall turnover of amphibian assemblages (solid line) and the five most responsive species for each variable across the Great Barrier Reef watershed. The file ‘Species_fits_and_variable_importance.xlsx’ indicates the species represented by each the remaining five lines.

**Appendix S27**

**Appendix S27.** The cumulative importance of the four most influential predictors in predicting the overall turnover of bird assemblages (solid line) and the five most responsive species for each variable across the Great Barrier Reef watershed. The file ‘Species_fits_and_variable_importance.xlsx’ indicates the species represented by each the remaining five lines.
